# High-throughput amplicon sequencing datasets of the metacommunity DNA of the gut microbiota of Zebrafish *Danio rerio* fed diets with differential quantities of protein and fat contents

**DOI:** 10.1016/j.dib.2022.108313

**Published:** 2022-05-23

**Authors:** George B. H. Green, Michael B. Williams, Sophie B. Chehade, Casey D. Morrow, Stephen A. Watts, Asim K. Bej

**Affiliations:** a Department of Biology, The University of Alabama at Birmingham, 1300 University Blvd., Birmingham, AL 35294, United States of America; b Department of Cell, Developmental and Integrative Biology, The University of Alabama at Birmingham, 1918 University Blvd., Birmingham, AL 35294, United States of America

**Keywords:** Metagenome, Animal nutrition, QIIME2, High-throughput sequencing, Animal model, Laboratory-formulated diet

## Abstract

In this paper, we present high-throughput amplicon sequence (HTS) datasets of the gut microbiota of male and female Zebrafish *Danio rerio* fed diets consisting of sub-optimal and above-optimal quantities of proteins and fats. The HTS datasets were generated using an Illumina MiSeq targeting the V4 hypervariable segment of the 16S rRNA gene. The raw sequence reads were quality checked, demultiplexed into FASTQ files, denoised using DADA2 (q2-dada2 denoise-paired), and subsampled. Taxonomic ids were then assigned to amplicon sequence variants (ASVs) against the silva-138-99-nb-classifier for taxonomic output using the Quantitative Insights Into Microbial Ecology (QIIME2 v2021.4). The resultant taxa list was generated at the phylum level to confirm the applicability of the HTS dataset using the "qiime taxa collapse" command. These HTS datasets of the metagenome can be accessed through the BioSample Submission Portal (https://www.ncbi.nlm.nih.gov/bioproject/) under the BioProject IDs PRJNA772302 and PRJNA772305.

## Specification Table

**Table:** 

Subject	Biology
Specific subject area	Metagenomics and bioinformatics
Type of data	Figures and Tables
How data was acquired	Illumina MiSeq platform with 250 paired-end kits.
Data format	Raw HTS FASTQ Format
Experimental factors	Laboratory aquaculture male and female Zebrafish (*Danio rerio*) fed diets with various combinations of sub-optimal and above-optimal quantities of proteins and fats.
Data source location	UAB Department of Biology, 1300 University Blvd., Birmingham, AL 35294, USA. Microbial metacommunity DNA was prepared and sequenced at the UAB Department of Genetics, Heflin Center Genomics Core, School of Medicine, the University of Alabama at Birmingham, 705 South 20th Street, Birmingham, AL 35294, USA. (33.2140° N, 87.5391° W).
Data	Raw data corresponding to the 16 samples are available at the NCBI's BioSample database following this link: 1. Beige Diet - above optimal protein and sub-optimal fat: PRJNA772305 https://www.ncbi.nlm.nih.gov/sra?linkname=bioproject_sra_all&from_uid=772305 2. Red Diet - suboptimal protein and above optimal fat: PRJNA772302 https://www.ncbi.nlm.nih.gov/sra?linkname=bioproject_sra_all&from_uid=772302

## Values of the Data

• These HTS datasets will help expand our knowledge about the relationship between the laboratory-formulated diets and the gut microbial community compositions in *Danio rerio* and potentially other model vertebrate animals used in various laboratory studies.
• The raw metagenome data manifested restructuring of the microbial community composition in gender-specific *Danio rerio* fed a laboratory-formulated diet with variable quantities of protein and fat.
• Access to the raw HTS data will allow researchers to analyze and use them for their own scientific studies.

## 1 Data

**Fig. 1:**
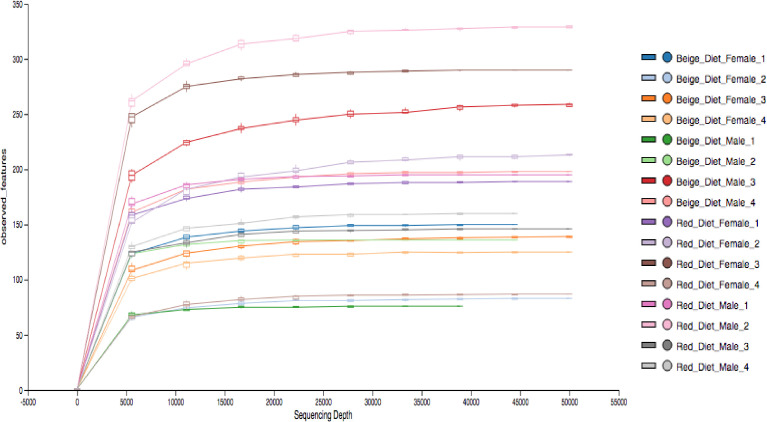
Rarefaction curve analysis was performed on the high-throughput sequence (HTS) datasets displaying observed features (y-axis) plotted against the number of sequences (x-axis) per sample. Observed features were generated via QIIME2 (v2021.4). Data was plotted using the view command of the QIIME2 tool.

**Fig. 2:**
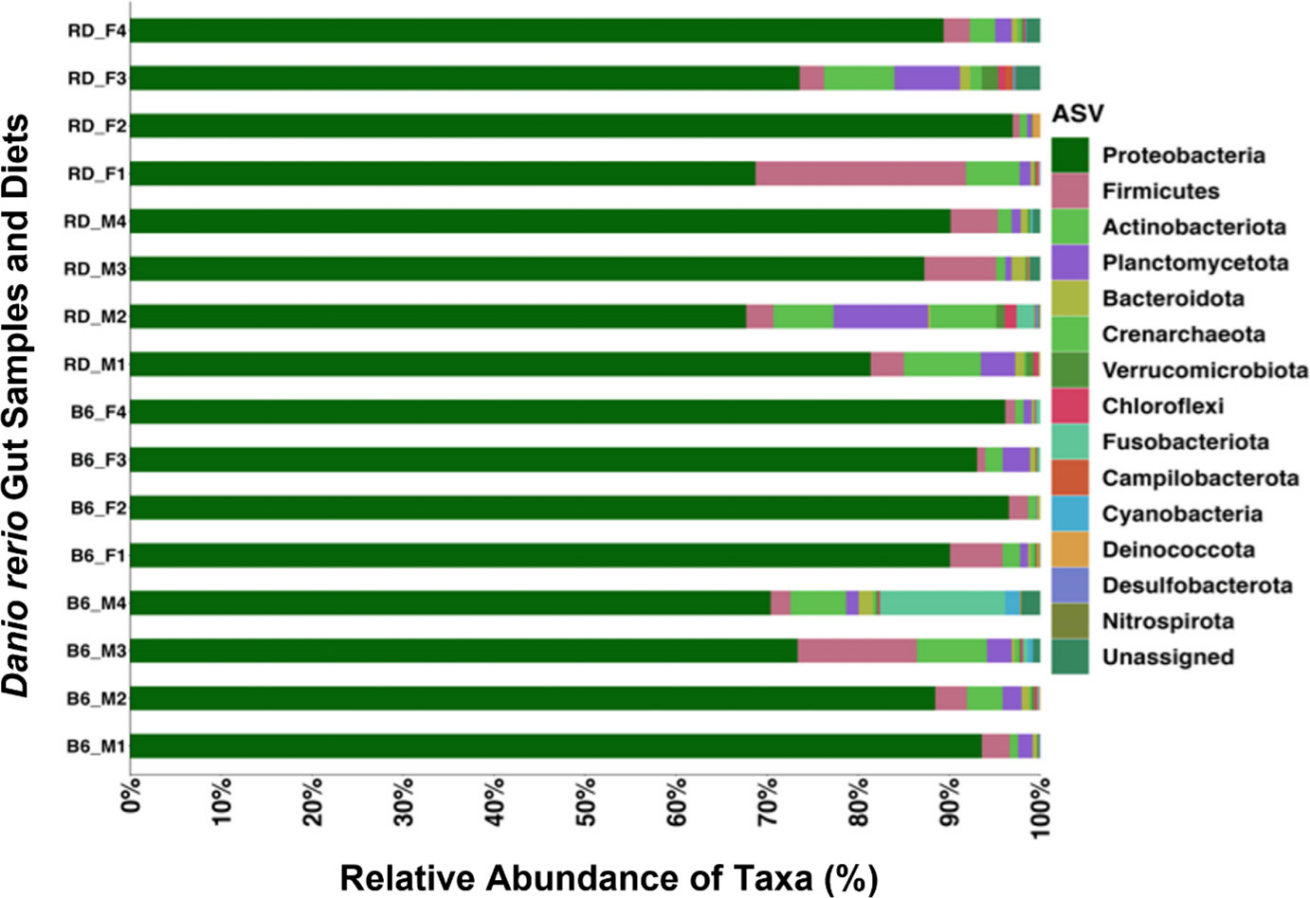
The horizontally stacked column bar plot shows the highest abundance of the top 15 Phyla across all *Danio rerio* gut samples (*n* = 16). The taxonomic identities were established utilizing the SILVA v138 (silva-138-99-nb-classifier.qza) database, determined by the Quantitative Insights into Microbial Ecology (QIIME_2, v2021.4), and visualized using R (ggplot package). Sample designations are as follows: B6_M = above optimal quantities of protein (47.29%) and fat (11.04%) fed Male *Danio rerio*; B6_F = above optimal quantities of protein (47.29%) and fat (11.04%) concentrations fed Female *Danio rerio*; RD_M = sub-optimal quantities of protein (34.08%) and fat (4.61%) fed Male *Danio rerio*; RD_F = sub-optimal quantities of protein (34.08%) and fat (4.61%) fed Female *Danio rerio*.

The HTS data presented in this manuscript describes the gut microbial community composition of a commonly used laboratory experimental vertebrate animal zebrafish *(D. rerio)* males (*n*=8) and females (*n*=8) fed diets with (1) above optimal protein (47.29%) in combination with suboptimal fat (4.61%); and (2) suboptimal protein (34.08%) with above optimal fat (11.04%). We used Illumina MiSeq to generate the HTS dataset by targeting the V4 hypervariable segment of the 16S rRNA gene amplicon [1]. The rarefaction analysis of the HTS data from each sample indicated reaching saturation when constructed at 3% sequence variation (Fig. 1), thus confirming the quality of the data. The microbial community composition determined from the quality checked HTS datasets at the phylum level showed, in general, high abundances of Proteobacteria ranging from 81.4% to 94% and Firmicutes ranging from 4.95% to 7.3% across all samples. In contrast, Actinobacteria represented higher abundances in female *D. rerio* (4.3%) fed diets consisting of above optimal quantities of protein and fat contents compared to the sub-optimal quantities of fat and protein fed diets (1.4%) (Fig. 2). For ease of description, the diets with various quantities of proteins and fats were assigned specific color codes (Table 1). The distribution of the top 15 phyla and the relative abundances across male and female *D. rerio* fed various diets presented in Table 2 confirm the quality and applicability of the datasets presented in this manuscript.

**Table 1:** The designation of the Zebrafish (*Danio rerio*) diet types and representative color codes were used in this study.

Diet Type	Color Code
Above optimal quantities of protein (47.29%) in combination with suboptimal fat (4.61%) fed female *D. rerio*	Beige_female: SAMN21857057 - SAMN21857064 (PRJNA772305)
Above optimal quantities of protein (47.29%) in combination with suboptimal fat (4.61%) fed male *D. rerio*	Beige_male: SAMN21857057 - SAMN21857064 (PRJNA772305)
Suboptimal quantities of protein (34.08%) in combination with above optimal fat (11.04%) fed female *D. rerio*	Red_female: NCBI BioSample Ids: SAMN21857065 - SAMN21857072 (PRJNA772302)
Suboptimal quantities of protein (34.08%) in combination with above optimal fat (11.04%) fed female *D. rerio* (PRJNA772302)	Red_male: NCBI BioSample Ids: SAMN21857065 - SAMN21857072

**Table 2:** Statistical analysis of the representative sequences aligned to SILVA v138 (silva-138-99-nb-classifier.qza). Taxonomic assignments were performed using QIIME2 (v2021.4), and percentages were calculated across all *Danio rerio* samples (*n*=16). The top 15 phyla were selected based on total relative abundance. (ASV = Amplicon Sequence Variants).

ASV	Beige_Male (%)	Beige_Female (%)	Red_Male (%)	Red_Female (%)
Proteobacteria	81.45	94	81.6	82.13
Firmicutes	5.48	2.48	4.95	7.38
Actinobacteriota	4.65	1.4	4.38	4.3
Planctomycetota	1.95	1.23	3.98	2.68
Fusobacteriota	3.63	0.2	0.58	0
Crenarchaeota	0.3	0.15	1.98	0.45
Unassigned	0.7	0	0.5	1.03
Bacteroidota	0.78	0.33	0.8	0.53
Verrucomicrobiota	0.23	0.08	0.5	0.58
Chloroflexi	0.18	0.08	0.48	0.3
Cyanobacteria	0.55	0	0.08	0.08
Deinococcota	0.05	0.05	0.05	0.23
Campilobacterota	0	0	0.03	0.2
Nitrospirota	0.03	0.03	0.05	0.03
Desulfobacterota	0.03	0	0.03	0.05

## 2 Experimental Design, Materials and Methods

### 2.1 Sample collection

*D. rerio* embryos (AB strain) were collected randomly from a mass spawning of males and females. The embryos were transferred to Petri dishes, and the freshly hatched larvae polycultured in static tanks with rotifers *Brachionus plicatilis* [2]*.* Rotifers were then fed a blend of six microalgae (RotiGrow Plus, Reed Mariculture, Inc., Campbell, CA, USA). After 11-days post-fertilization (dpf), the animals were fed Stage I Artemia Nauplii (INVE Aquaculture INC., Salt Lake City UT, USA) until 28-dpf. All adult animals maintained in the tanks were then combined and randomly distributed in 2.8L tanks (14 fish per tank, *n* = 10 tanks per treatment). Tanks were maintained on a recirculating aquatic system (Aquaneering, Inc., San Diego, California, USA) under the flow rate of *ca.* two full tank exchanges per hour. Water was maintained at pH 7.4, 28 °C, and 1500 µS/cm. Colorimetric tests were used once weekly to ensure appropriate levels of nitrogen, nitrite, and nitrate. Each tank was then assigned standard or differential protein and fat-containing diets to start the feeding experiment. Throughout the experiment, the tanks were maintained on the same recirculating system and cleaned and placed in a new position on the recirculating rack system every two weeks. The tanks were siphoned on alternate days to remove unconsumed food and debris. The zebrafish were maintained under a 14-hour light/10-hour dark cycle. At the termination of the 14-week feeding, four males and four females from each dietary regimen had whole guts (stomach and intestine) dissected out and flash-frozen in liquid nitrogen before being transferred to a −80 °C ultralow temperature freezer until used for microbiome analysis.

### 2.2 High-throughput sequencing

The community DNA from *D. rerio* whole guts was purified using the Zymo Research kit (Irvine, CA, USA) per the manufacturer's instructions. Purified DNA was subjected to quantification and purity assessment using a NanoDrop (Nanodrop One C, Thermo Fisher Scientific, 5225 Verona Rd., Madison, WI, USA) and sent to UAB Microbiome Resource Center for high-throughput sequencing (HTS). The HTS was performed using Illumina MiSeq and 250 bp paired-end kits (Illumina, Inc., San Diego, CA, USA) targeting the V4 hypervariable region of the 16S rRNA gene amplicons. The resultant sequences were demultiplexed, FASTQ formatted, and deposited to the National Center for Biotechnology Information (NCBI) Sequence Read Archive (SRA) under Bioproject #PRJNA772305 and #PRJNA772302 for the Beige_diet and Red_diet sample, respectively. The pair-end sequence data for the respective diet groups is available under the following NCBI BioSample Ids: SAMN21857065 - SAMN21857072 (Red_diet) and SAMN21857057 - SAMN21857064 (Beige_diet) (Table 1). The subgroups of the experimental *D. rerio* were designated in this study as follows: suboptimal quantities of protein and fat-fed female *D. rerio* (Beige_female, *n* = 4), and sub-optimal concentrations of protein and fat-fed male *D. rerio* (Beige_male, *n* = 4); and for the high protein low-fat group were labeled as follows: above optimal protein and fat concentrations female *D. rerio* (red_female, *n* = 4), and optimal protein and fat concentrations male *D. rerio* (red_male, *n* = 4) (Table 1)

### 2.3 Taxonomic classification

The HTS datasets were first demultiplexed into FASTQ files and then imported into Quantitative Insights Into Microbial Ecology (QIIME2). Using the cassava 1.8 paired-end demultiplexed fastq format for subsequent analyses [3]. The raw data were then quality checked using "qiime demux summarize" function. Denoising was performed via DADA2 (q2-dada2 denoise-paired) [4]. The representative sequences (rep-seqs) were generated via "q2- feature-table tabulate-seqs" command. The mafft embedded program aligned the amplicon sequence variants (ASVs) [5], and the output was piped into fasttree2 (q2-phylogeny) [6]. Alpha and Beta diversity statistics were generated via the "core-metrics-phylogenetic" command [7]. The samples were rarefied to a minimum of 41,356 sequences per sample (subsampled without replacement). Taxonomic identifications (ids) represented the respective sequences assigned to ASVs against the Silva-138-99-nb-classifier [8]. The taxonomy generated was collapsed (1-7) using "qiime taxa collapse" command [3] and listed at the phylum level (Fig. 2) to confirm the applicability of the HTS datasets for the *D. rerio* gut microbiome studies.

## Ethics Statement

All animal experiments were conducted following the guidelines approved by the animal care and use under the Institutional Animal Care and Use Committee Permit IACUC-21893; 2021-Nov-2022 (S.A.W.), the University of Alabama at Birmingham, and the laboratory certified by the American Association for Accreditation of Laboratory Animal Care (AAALAC) International. All experiments in this study followed the Animal Research: Reporting of In Vivo Experiments (ARRIVE) guidelines that are applicable to the zebrafish model.

## CRediT Author Statement

**George B. H. Green:** Formal data analysis, writing the original draft of the manuscript; **Casey D. Morrow:** High-throughput sequencing, review and editing manuscript; **Michael B. Williams and Sophie B. Chehade:** Animal experiments, editing manuscript; **Stephen A. Watts:** experiment design, supervise the animal experiments under the IACUC (UAB), ARRIVE and AAALAC animal use and feeding compliances, editing manuscript; **Asim K. Bej:** overall supervision of the study, data interpretation, writing and editing the manuscript.

## Declaration of Competing Interest

The authors declare no known competing financial interests or personal relationships that could have influenced the work reported in this paper.

## Data Availability

Metagenome datasets of gut microbiome of zebrafish D. rerio fed Red Diet - suboptimal protein and above optimal fat (Original data) (NCBI). Metagenome datasets of gut microbiome of zebrafish D. rerio fed Beige Diet - above optimal protein and sub-optimal fat and Red Diet - suboptimal protein and above optimal fat (Original data) (NCBI).
